# Olfactory and Gustatory Function before and after Laparoscopic Sleeve Gastrectomy

**DOI:** 10.3390/medicina57090913

**Published:** 2021-08-31

**Authors:** Cecilia Berro, Alfonso Luca Pendolino, Mirto Foletto, Maria Cristina Facciolo, Pietro Maculan, Luca Prevedello, Diletta Giulia Giuntoli, Bruno Scarpa, Chiara Pavan, Peter J. Andrews, Giancarlo Ottaviano

**Affiliations:** 1 Department of Neurosciences, Otolaryngology Section, University of Padova, 35128 Padova, Italy; cecilia.berro@studenti.unipd.it (C.B.); info@otorinolaringoiatrafacciolo.it (M.C.F.); dggiuntoli@gmail.com (D.G.G.); giancarlo.ottaviano@unipd.it (G.O.); 2 Department of ENT, Royal National ENT & Eastman Dental Hospitals, London WC1E 6DG, UK; peter.andrews@ucl.ac.uk; 3 Ear Institute, University College London, London WC1X 8EE, UK; 4 Centre for Mechanics of Biological Materials, University of Padova, 35128 Padova, Italy; mirto.foletto@unipd.it; 5 IFSO Bariatric Centre of Excellence, Padova University Hospital, 35128 Padova, Italy; luca.preve@sanita.padova.it; 6 Department of Cardiac Thoracic Vascular Sciences and Public Health, University of Padova, 35128 Padova, Italy; pietro.maculan@unipd.it; 7 Department of Statistical Sciences and Department of Mathematics Tullio Levi-Civita, University of Padova, 35128 Padova, Italy; scarpa@stat.unipd.it; 8 Department of Psychiatry, University of Padua, 35128 Padua, Italy; chiara.pavan@unipd.it

**Keywords:** smell, taste, olfactory threshold, bariatric surgery, sleeve gastrectomy, taste quality, MMSE

## Abstract

*Background and Objectives*: Bariatric surgery is the gold standard for the treatment of morbid obesity, and current evidence suggests that patients undergoing surgery can show changes in their sense of taste and smell. However, no definitive conclusions can be drawn given the heterogeneity of the studies and the contrasting results reported in the literature. *Materials and Methods*: We enrolled 18 obese patients undergoing laparoscopic sleeve gastrectomy (LSG) and 15 obese controls. At baseline (T0) and 6 months after enrollment/surgery (T1), both groups underwent Sniffin’ Sticks and whole mouth test. Post-operative qualitative taste variations were also analyzed and SNOT-22, VAS for taste and smell, and MMSE were administered. *Results*: An improvement in the olfactory threshold was observed in the treatment group (*p* = 0.03) at 6 months. At multivariate analysis, the olfactory threshold differences observed correlated with MMSE (*p* = 0.03) and T0 gustatory identification (*p* = 0.01). No changes in sense of taste were observed between the two groups at 6 months, even though nine subjects in the treatment group reported a worsening of taste. This negatively correlated with age (*p* < 0.001), but a positive marginal correlation was observed with the olfactory threshold difference between T0 and T1 (*p* = 0.06). *Conclusions*: Olfaction can improve after LSG, and this seems to be the consequence of an improved olfactory threshold. Although we did not observe any change in gustatory identification, food’s pleasantness worsened after bariatric surgery.

## 1. Introduction

The proportion of obese people is constantly increasing worldwide [1]. Bariatric surgery is considered the gold standard for the treatment of morbid obesity, leading to sustained body weight reduction, improvements in metabolic health and comorbidities, and decreased mortality [2]. The Roux-en-Y gastric bypass (RYGB) and sleeve gastrectomy (SG) represent the most effective long-term treatments for severe obesity and, respectively, account for 17.8% and 59.4% of all bariatric procedures [3].

However, it is well known that the weight loss observed following bariatric surgery is not only linked to a smaller food intake or to additional malabsorptive effects in RYGB, but instead to multiple and more complex mechanisms [4,5]. Current evidence confirms that patients undergoing bariatric surgery can show changes in their sense of taste and smell, appetite, and food preferences [6,7,8]. In a survey conducted on 154 patients, Zerrweck et al. [9] found that taste and olfactory changes were present in a vast majority of patients who underwent bariatric surgery, regardless of the type of procedure. Moreover, the link between the olfactory and gustatory systems is well established [10], and olfactory dysfunction results in a decreased flavor perception in up to 69% of cases [11]. Additionally, smell function can change during hunger, and a previous study found that the olfactory threshold is better when fasting [12]. Therefore, alterations in either the sense of taste and/or smell can contribute to changes in eating behavior and subsequent weight loss following bariatric surgery.

Nevertheless, studies adopting validated taste/olfactory tests to evaluate post-operative changes still remain limited and, even though the majority of authors seem to agree on gustatory improvements following bariatric surgery, contrasting results have been reported with regard to the olfactory function. In particular, whereby olfaction is measured by means of Sniffin’ Sticks (S’S)—a validated psychophysical test—and a composite TDI score is calculated (which thus implies an evaluation of the threshold, discrimination, and identification sub-tests), conflicting results have been reported on which of the S’S sub-tests improves after surgery.

In the present study, we investigated olfactory and gustatory functions in a group of obese patients before and 6 months after laparoscopic sleeve gastrectomy (LSG) by means of patients reported outcome measures [PROMs—namely, visual analog scale (VAS) for smell and taste, mini-mental state examination (MMSE), and a 22-item sinonasal outcome test (SNOT-22)] and psychophysical tests [S’S and whole mouth test (WMT)]. A group of obese patients not undergoing bariatric surgery served as a control group. Additionally, we evaluated patients’ pre-operative cognitive status in a bid to avoid unintentionally including subjects with smell and/or taste alterations due to cognitive deficits.

## 2. Materials and Methods

This study was conducted in accordance with the Helsinki Declaration and approved (09/2011) by the Padua Otolaryngology Section’s ethical committee (prot. n. 2244/2011). Data were examined in agreement with the Italian privacy and sensible data laws (D.Lgs 196/03) and the otolaryngology section internal regulation. Written informed consent was obtained from all patients prior to their inclusion in the study.

Between September 2016 and June 2017, adult obese patients undergoing LSG at the Obesity Centre of the University Hospital of Padua were enrolled. A control group of obese subjects submitted to LSG but still waiting for surgery was also included.

All subjects initially received an MMSE [13] and a SNOT-22 [14], respectively, for cognitive status and sinonasal symptom evaluations. Patients with an MMSE score lower than 18, suggestive of possible severe cognitive deficit, or subjects with a SNOT-22 score higher than 22, indicative of possible sinonasal pathology, were excluded [15]. Other exclusion criteria were diabetes in therapy with insulin [16], hypothyroidism, tobacco smoking (current or in the last 5 years), history of allergic/non-allergic rhinitis or chronic rhinosinusitis, previous sinonasal surgery, history of head trauma or post-viral olfactory loss, and previous chemotherapy/radiotherapy to head and neck [17].

At T0, enrolled subjects satisfying the MMSE and SNOT-22 minimal scores underwent smell evaluation by means of extended S’S [including the odor threshold (T), discrimination (D), and identification (I) sub-tests] and a screening gustatory identification test (WMT). The latter consisted of four taste solutions (sweet, sour, salty, bitter) sprayed into the oral cavity at a suprathreshold concentration (sweet: 0.4 g/mL sucrose; sour: 0.3 g/mL citric acid; salty: 0.25 g/mL sodium chloride; bitter: 0.006 g/mL quinine hydrochloride). Distilled water was used as a solvent [18]. The test score ranges between 0 and 4. In order to know patients’ subjective chemosensory perceptions, each participant was asked to complete a VAS for taste (tVAS) and smell (sVAS) (0 corresponded to not affected and 10 to the worst thinkable situation) [19]. In particular, for tVAS, the question was “How would you rate your basic taste (sweet, sour, salty, bitter) from 0 to 10?”, while for sVAS, the question was “How would you rate your sense of smell from 0 to 10?”. The same tests were performed in the control group at baseline. In the treatment group, the LSG was performed starting at 4 cm from the pylorus over a 40 Fr bougie, with an average of 5 60 mm reloads without oversuturing the staple line. A methylene blue dye test was carried out intraoperatively. Drainage was liberally placed and removed on post-operative day 2, whenever output was less than 50 mL/day and oral methylene blue dye was negative.

Six months after the first evaluation (T1), all tests (apart from MMSE and SNOT-22) were repeated both in the treatment and in the control groups. In addition, at T1, all subjects in the treatment group were also asked whether they had noted any change in the pleasantness of food or its palatability (namely flavor perception/fine taste) following LSG. Answers were scored as 1 (I feel no change), 2 (I feel a deterioration in the qualitative perception of tastes), and 3 (I feel an improvement in the qualitative perception of tastes) [20]. All chemosensory tests were conducted during the morning, and patients were asked to not eat, drink coffee, smoke, or brush their teeth up to 2 hours before measurements. The tests were performed in a well-ventilated and odorless room. Body mass index (BMI—kg/m^2^), calculated by dividing patient’ weight in kilograms by patient’ height in meters squared, was measured at T0 and T1 for all subjects.

### Statistical Analysis

A Welch two sample *t*-test was used to compare the differences in TDI, threshold, discrimination and olfactory identification, and gustatory identification in the time interval T0–T1 between the two groups. A multivariate linear regression model was used to evaluate which variables influenced significant differences. A simple univariate regression model allowed us to evaluate the relationship of qualitative changes after surgery with the olfactory threshold difference in the time interval T0–T1 and with age. A *p*-value < 0.05 was considered statistically significant. Values in the range of 0.10 > *p* ≥ 0.05 were considered as indicative of a statistical trend. R, a language and environment for statistical computing (R Foundation for Statistical Computing, Vienna, Austria), was used for all statistical analyses.

## 3. Results

In total, 33 adult subjects (18 patients and 15 controls) were enrolled. Both groups had comparable characteristics at the baseline. Demographic details and olfactory and gustatory scores for the two groups at T0 and T1 are reported in Table 1.

**Figure 1:**
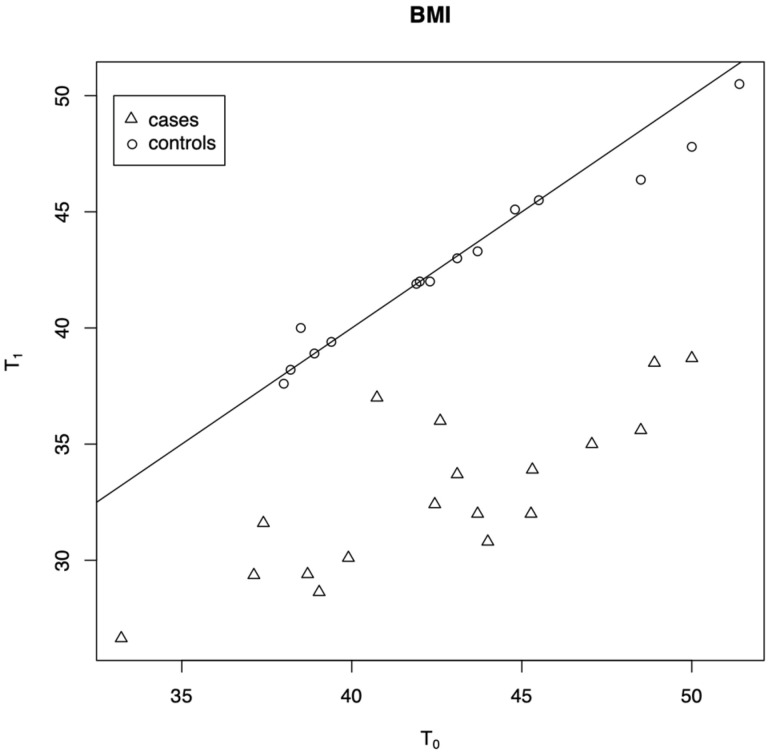
Scatter plot of the variables for BMI at T0 and T1.

A significant decrease in BMI score between T0 and T1 was observed in the treatment group following LSG (*p* < 0.0001), while no significant changes were found in the control group at the two separate examinations (*p* = 0.726) (Figure 1). At T0, only one patient (1/18; 5.6%) in the treatment group reported a lower score at sVAS, while none of them reported any change in their sense of taste (tVAS). At T1, two subjects (2/18; 11.1%) reported a lower score either at sVAS and tVAS following LSG, while only one of them (1/18; 5.6%) reported a taste reduction at tVAS post-operatively. In the treatment group, food’s pleasantness worsened in nine patients (9/18; 50%) after LSG, remained unchanged in eight of them (8/18; 44.4%), and improved in only one subject (1/18; 5.6%). In the control group, none of the subjects experienced any changes in their sense of smell or taste (sVAS and tVAS) between T0 and T1.

**Figure 2:**
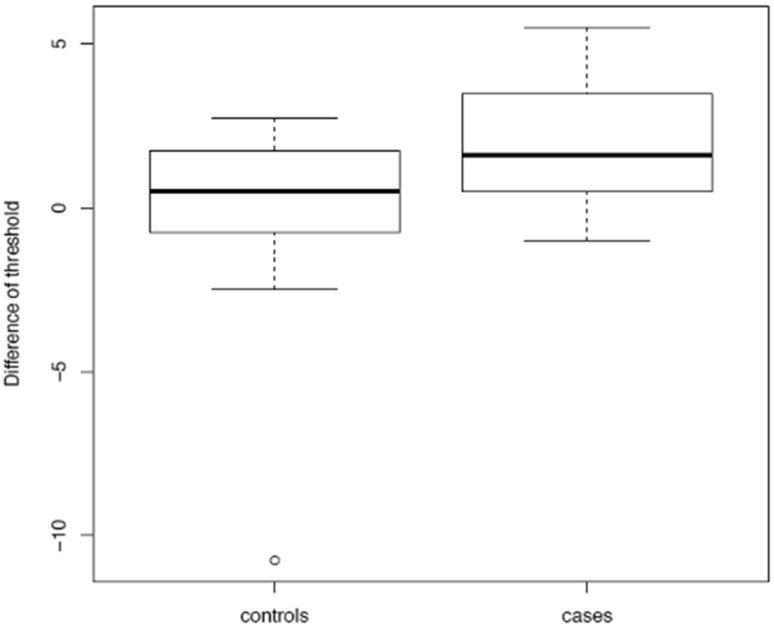
Boxplot illustrating the differences in threshold between T0 and T1 in the two groups.

No significant differences in the TDI score and the S’S sub-scores were found at T0 between the treatment and the control groups (*p* = 0.764). Similarly, no significant differences were found in the two groups between T0 and T1 in terms of TDI (*p* = 0.223), D (*p* = 0.386) and I (*p* = 0.217) scores, even though a significant improvement in the T score was observed in the treatment group at T1 when compared to T0 (*p* = 0.032) (Figure 2). No significant improvement in gustatory identification, as measured by means of a WMT, was observed following LSG (*p* = 0.9).

**Figure 3:**
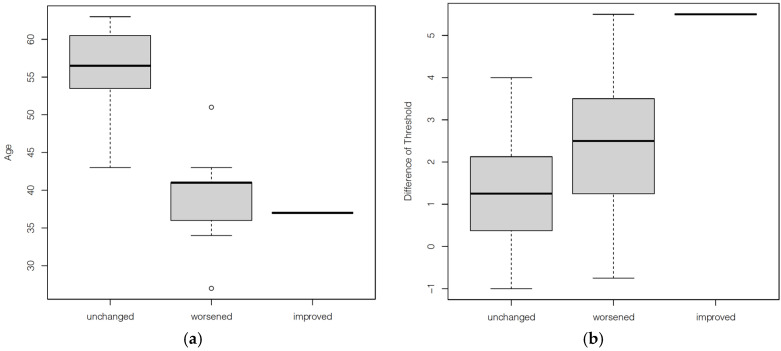
Boxplot showing qualitative gustatory variations in relation: (**a**) to age and (**b**) to the threshold difference between T0 and T1. Qualitative variations in taste were unchanged for 8 patients, were worsened for 9, and improved for only 1 patient.

In the multivariate analysis, performed to evaluate any possible correlation between olfactory threshold (T score) differences at T0 and T1 and all the available variables, a significant correlation was found between MMSE (*p* = 0.029) and T0 gustatory identification (*p* = 0.012) (Table 2). An analysis of the qualitative variations in taste (food’s pleasantness) following LSG showed a statistically significant inverse relationship with age (*p* < 0.001) (Figure 3a) and a marginal one with the variation in T score between T0 and T1 (*p* = 0.058) (Figure 3b).

## 4. Discussion

In the present prospective and controlled study, we demonstrated that, even though the total capacity for smelling (TDI score) did not change in either the treatment or the control group 6 months after the first evaluation, olfactory threshold significantly improved in the treatment group following LSG. However, conflicting results have been reported in the past by other authors. Although two previous studies confirmed an improvement in the total TDI score in patients undergoing SG [7,8], Holinski et al. [7] observed that this was mainly due to an improvement in odor discrimination, while Hanci et al. [8] found a significant improvement in all the three sub-test scores. Richardson et al. [21] and Zerrweck et al. [6] studied smell identification before and after gastric bypass but reached opposite conclusions. More recently, Melis et al. [22] observed improved smell identification scores following different bariatric procedures (including SG, RYGB or mini gastric by-pass). However, these results [6,21,22] are not strictly comparable with each other as they use different identification tests in their studies (cross cultural smell identification test, the pocket smell test, or S’S). The improvement in the olfactory threshold in our study may have been influenced by the low number of subjects included. Nevertheless, a similar result was recently reported by Pisarska-Adamczyk and colleagues [23] in a controlled study involving 53 patients undergoing bariatric surgery (LSG or laparoscopic RYGB) and 30 controls, and post-operative olfactory function was measured shortly after surgery (one day post-op). Similarly, Jurowich and colleagues [24], when comparing TDI scores in 15 RYGB and 15 SG patients pre- and post-operatively, found an increase in the threshold in the SG group only, with no changes to discrimination and identification in either group post-operatively.

Olfactory identification, discrimination, and threshold evaluate different aspects and areas of the olfactory system. While the olfactory threshold better describes peripheric olfactory system activity, olfactory identification and discrimination seem to be more associated with cognitive functions [25,26]. Moreover, an improvement in the olfactory threshold after surgery could be linked to the fact that this is more sensitive to limited modifications in the olfactory function when compared to olfactory identification [27].

Taste changes following bariatric surgery have been widely described. According to a recent systematic review, reported changes in taste include an increase in sensitivity to sweet and fatty taste stimuli and a decrease in preference to sweet-tasting stimuli, which could contribute to the long-term maintenance of weight loss post-operatively [28]. Additionally, in a case–control investigation conducted in 100 obese women, Hubert et al. [3] concluded that patients who are successful in weight loss 1 year post-bariatric surgery are more prone to report either improvements or alterations in their taste and flavor perceptions. Interestingly, using functional magnetic resonance imaging (fMRI), Wang et al. [29] found a significant change in brain activation in the reward system of obese patients after RYGB for salty tastes but not for sweet tastes when compared to the same individuals’ presurgical activation levels. In our study, we did not observe any improvement in taste identification (using the WMT) following LSG, while food’s pleasantness worsened post-operatively in the majority of patients (50%; 9/18), with only one of them reporting an improvement. Interestingly, changes in flavor perception correlated with patients’ age, whereby younger patients reported higher flavor alterations. Moreover, this also correlated marginally with olfactory threshold improvement following LSG. This could be explained by the fact that younger people, having a better taste, can perceive flavor alterations more easily than the older ones, though with a normal taste function. Another reason could be that, due to the fact that an olfactory threshold improvement was observed in younger patients, this improvement could have exposed these subjects to a higher odor sensitivity and a consequent alteration in their perception of flavor. Nevertheless, Holinski et al. [7], using the taste strips test, found an increased taste identification score in 44 patients 6 months after bariatric surgery (RYGB, SG or adjustable gastric banding). This was later replicated by Altun and collaborators [30] in 52 morbidly obese patients after LSG (3 months post-op) and by Melis et al. [22] in 51 patients undergoing different bariatric surgery procedures (including SG, RYGB, or mini gastric by-pass), always using the same test. Moreover, Altun et al. [30] observed an increase in taste sensitivity in all patients for sweet and salty foods and in some patients regarding bitter and sour taste strips in those undergoing LSG. The discrepancy in the result found in our cohort (i.e., lack of an improvement in the identification score following LSG) could also be linked to the fact that we used a quick screening test for gustatory function whereby only the highest concentrations for each flavor were presented to patients. In this regard, we might have missed possible mild changes in the sense of taste post-operatively. Nevertheless, a significant decrease in patient thresholds for bitter and sour taste and a non-significant decrease in sweet and salty taste before and after gastric by-pass have also been reported by some authors [31]. Moreover, Tichansky et al. [32] found that 82% and 46% of patients undergoing gastric bypass and gastric banding, respectively, noted alterations in their taste of food or drink after surgery, with many patients developing aversions to certain foods or changing food preferences after surgery.

To our knowledge, this is the first study that considers the impact of cognitive status in the study of olfactory and taste modifications in patients undergoing bariatric surgery. Our multivariate analysis showed that both mental status (in terms of pre-operative MMSE) and gustatory identification at enrolment had a significant influence on the olfactory threshold modifications observed between T0 and T1. It is well known that a correlation exists between cognitive status and one’s sense of smell [26], and a previous study confirmed that MMSE is an independent variable that is able to influence the olfactory threshold [17]. In this regard, our results add important data to the available literature.

The exact mechanisms by which olfactory and gustatory changes following bariatric surgery occur still remain unclear. A recent systematic review found evidence that supports that reported changes in taste sensitivity and its relation to food preference may be partly due to intrinsic changes within the gustatory and olfactory systems following bariatric procedures [28]. A relationship between olfactory modulation and nutritional status has been suggested, at least in rodents [33]. Recently, a relationship between the olfactory system and the endocrine system, in terms of gherkin and leptin (peptides important in appetite control and obesity pathogenesis), has been demonstrated, as their receptors are expressed in the olfactory mucosa and regulated by starvation [34]. Moreover, a combination of gut hormone and central nervous system effects may account for the observed changes in sensory function after weight loss surgery [35,36]. Neurohormonal mechanisms could also be involved. Peptides found in taste cells, such as glucagonlike peptide-1 (GLP-1) and peptide tyrosine tyrosine (PYY), have been found to increase following LGBP and LSG [37,38]. Similarly, the CCK (cholecystokinin), NPY (neuropeptide Y), VIP (vasoactive intestinal peptide), and ghrelin have been shown to be affected to different degrees after bariatric surgery [39]. Harris and Griffin [40] speculated that vagal influences might be responsible for many of the changes after weight loss surgery, but this seems to be unlikely considering that the vagus nerve is routinely preserved during the course of bariatric surgical procedures. Gastroesophageal reflux, a common side effect of morbid obesity, as well as bariatric surgery (especially after SG), could contribute to a distorted sense of taste (dysgeusia) [41]. More recently, Melis et al. concluded that genetic factors, such as OBPIIa gene polymorphisms and heritable variations in PROP taste sensitivity, can play an important role in the bariatric surgery-induced changes in taste function [22].

The exact duration of these alterations is still unknown. Zerrweck et al. [9] found that, in several cases, taste and olfactory changes were still present during the first 2 months following bariatric surgery, while Harris et al. [40] concluded these could last for years.

## 5. Conclusions

Olfaction can improve after LSG, and it seems to be consequence of an improved olfactory threshold. Although we did not observe any change in gustatory identification, food’s pleasantness worsened after bariatric surgery. Therefore, patients must be routinely counselled about potential changes in taste and smell as part of their informed consent prior to surgery. Even if the appearance of these changes is unpredictable, olfactory and gustatory alterations can be long lasting.

## Figures and Tables

**Table 1:** Demographic details and olfactory and gustatory scores for the two groups at T0 and T1.

**Treatment Group (*n* = 18)**	**T0**			**T1**		
	**Mean**	**SD**	**Range**	**Mean**	**SD**	**Range**
**Age** (**year**)	46.4	10.6	27–63	-	-	-
**BMI** (**Kg/m^2^**)	42.6	4.5	32.2–50	32.9	3.5	26.6–38.7
**Identification**	12.4	1.5	10–15	12.1	1.4	10–14
**Threshold**	7.3	1.6	4.25–10.25	9.3	2.2	4.75–12.75
**Discrimination**	12.3	1.3	10–15	12.3	1.7	8–16
**TDI**	32	2.6	27.25–36.5	33.8	3.1	27.75–38.75
**Taste identification**	3.9	0.3	3–4	3.8	0.4	3–4
**MMSE**	29	0.78	28–30	-	-	-
**VAS smell**	0.06	0.24	0–1	0.06	0.24	0–1
**VAS taste**	0	0		0	0	
**Control Group** (***n* = 16**)	**T0**			**T1**		
	**Mean**	**SD**	**Range**	**Mean**	**SD**	**Range**
**Age** (**year**)	48.9	11.6	21–67	-	-	-
**BMI** (**Kg/m^2^**)	43.1	4.3	38–51.4	42.8	3.7	37.6–50.5
**Identification**	12.6	1.1	11–14	12.9	1.3	10–15
**Threshold**	8.6	2.4	7–15.25	8.4	2.5	4.5–14
**Discrimination**	11.7	1.4	9–14	12.2	1.4	10–15
**TDI**	32.9	2.7	31–40.25	33.5	2.4	32.5–40.75
**Taste identification**	3.9	0.26	3–4	3.9	0.26	3–4
**MMSE**	29	1	27–30	-	-	-
**VAS smell**	0	0		0	0	
**VAS taste**	0	0		0	0	

T0: first evaluation; T2: second evaluation after 6 months; SD: standard deviation; BMI: body mass index; VAS: visual analogue scale (0 = not affected, 10 = the worst thinkable situation); TDI: threshold + discrimination + identification; MMSE: mini-mental state examination.

**Table 2:** Multivariate linear regression analysis.

Coefficients	Estimate	Std. Error	T Value	Pr (>|t|)
(Intercept)	7.5148	14.5831	0.515	0.6109
Cases	−4.2924	2.5770	−1.666	0.1083
MMSE	1.0540	0.4547	2.318	0.0289 *
BMI T0	−0.1966	0.1172	−1.677	0.1061
Δ BMI	−0.4027	0.2399	−1.678	0.1057
Olfactory threshold T0	−0.4377	0.2943	−1.487	0.1495
TDI T0	−0.2902	0.2304	−1.260	0.2195
Taste identification T0	−3.8598	1.4202	−2.718	0.0118 *

T0: first evaluation; BMI: body mass index; Δ BMI: delta BMI; TDI: threshold + discrimination + identification; MMSE: mini-mental state examination. * Significant *p*-values. Level of significance *p* < 0.05.

## Data Availability

The data presented in this study are available on request from the senior author (G.O.). The data are not publicly available due to privacy reasons.
